# An Effective Siddha Management for Dermatosis Papulosa Nigra: A Case Report

**DOI:** 10.7759/cureus.61668

**Published:** 2024-06-04

**Authors:** Saravanasingh Karan Chand Mohan Singh, Aishwarya A, Siva Lakshmi S, Karthi Senthil, Ramamurthy Murugan

**Affiliations:** 1 Anatomy (Udal Koorugal), National Institute of Siddha, Chennai, IND; 2 General Medicine (Pothu Maruthuvam), National Institute of Siddha, Chennai, IND; 3 Pharmacology (Gunapadam), National Institute of Siddha, Chennai, IND; 4 Pathology (Noi Naadal), National Institute of Siddha, Chennai, IND

**Keywords:** dermatosis papulosa nigra, seborrhoeic keratoses, single case report, pachaieruvai, siddha management

## Abstract

Dermatosis papulosa nigra (DPN) is a noncancerous skin growth that is classified as a subtype of seborrhoeic keratoses. DPN is predominantly found in individuals with darker skin tones, namely, those with Fitzpatrick skin types III-VI. This condition primarily affects women of Asian or African American descent. The non-availability of accurate management for this illness presents a challenge to the medical fraternity. Electrodesiccation, laser therapy, and surgery offer expensive solutions. Siddha external medicine provides a solution through appropriate methods. A 70-year-old woman with Fitzpatrick skin type V appeared with many hyperpigmented papules on the malar region that had been present for five years. The dermatological examination revealed the presence of several brown papular lesions, which were particularly pronounced in the malar areas. The dimensions of the lesions typically varied from 1 to 5 mm; however, in the malar area, their size extended up to 1 cm. The Siddha formulation *Pachaieruvai* was administered externally for five consecutive days. While undergoing treatment, the patient experienced mild pain, burning, slight redness, and swelling in the area where *Pachaieruvai *was applied. These symptoms persisted for about an hour during and after the procedure but disappeared within 24 hours with the use of coconut oil. The evaluation of treatment response was determined using the recognised quartile grading methodology. During the first-week follow-up appointment after the last treatment, significant improvements were observed in the cheek lesions, particularly in four large lesions.Throughout the treatment, the patient may encounter mild discomfort, a burning sensation, slight redness, and swelling at the administration site of *Pachaieruvai*. These reactions are positive indicators of disease regression. No adverse symptoms and recurrence were observed during the follow-up. This research specifically examines the Siddha approach to managing DPN. Based on the findings and observations, it can be concluded that Siddha medicine is effective in treating DPN.

## Introduction

Dermatosis papulosa nigra (DPN) is a harmless skin growth that is classified as a type of seborrhoeic keratoses. DPN is predominantly found in individuals with darker skin tones, namely, those with Fitzpatrick skin types III-VI. It is more prevalent in women of Asian or African American descent. Individuals of African heritage have the most significant occurrence of DPN, with a documented incidence ranging from 10% to 30%. Incidence rates of DPN are approximately 70% in African-American (AA) individuals and 40% in African individuals. Furthermore, it has been observed that DPN has a higher prevalence in women, occurring nearly twice as often as in men [1]. DPN can manifest on various areas of the face, neck, chest, and back; however, they are commonly characterised as painless brown to dark brown bumps that are found on the cheeks and around the eyes. The lesions typically range in size from 2 to 10 mm and can have either a filiform or sessile shape. The size and number of DPN may increase as one gets older, even if this condition is not harmful. DPN have been observed to negatively impact patients' quality of life and are considered unattractive due to their tendency to appear on the face, head, and neck. Due to their preference for the face, head, and neck, DPNs can be aesthetically unpleasing and have been found to have a detrimental effect on patients quality of life. Furthermore, the physical discomfort associated with DPN can also contribute to decreased quality of life. It is essential for individuals with this skin condition to seek proper medical care and support to manage both the physical and emotional aspects of the condition [2]. In the field of biomedicine, DPN are treated using various methods, such as topical keratolytic, electrofulguration, liquid nitrogen cryotherapy, laser vaporisation, and intra-lesioned chemotherapy, in order to completely remove them. Nevertheless, none of these comprehensive treatment approaches have consistently diminished the growth or thwarted the reappearance of DPN [3].

Siddha advocates a comprehensive strategy for treating DPN, which includes topical use of *kaaram, suttigai, seelai,* or surgical removal. *Kaara maruthugal* are alkaline substances derived from the ash of medicinal herbs, metalloids, dew droplets, and other sources. Siddha physicians widely use *kaaram*, in the form of powder, paste, or aqueous solution, for local treatment to effectively treat DPN. Nevertheless, these techniques necessitate many sessions and a longer time frame to eliminate the DPN, with restrictions on their effectiveness for smaller DPN. In this study, we provide an initial report on the treatment of DPN in a single patient utilising *kaaram-Pachaieruvai*​​. Siddha medicine is categorised as internal medicine, known as *Aga marundu* 32, and external medicine, known as *Puramarundu* 32. *Puramarunthugal*, commonly known as external medicine, encompasses several types of medications and specific applications, including nose, ocular, and ear drops. It also involves treatments, such as leech application, ointment usage, and chemical cauterization. The classification of this substance consists of 32 distinct categories, including *kattu *(bandage), *pattru* (poultice), *poochu* (liquid application), *kalimbu* (ointment), *seelai* (medical gauze), *kaaram* (chemical cautery), and others. *Kaaram*, also known as chemical cautery, is the application of medicated caustic medicines to remove undesired development in various conditions, such as external haemorrhoids, fistulas, non-healing ulcers, granulomatous ulcers, abscesses, toad skin, and tumours accompanied by itching. As per Siddha literature, *kaaram* refers to the method of using medicinal caustic chemicals to treat external growths.

This case report signifies the inaugural instance in the history of dermatology where the administration of *kaaram* medicine has effectively treated DPN. This medication is specifically intended for those with viral warts, moles, accessory tragus, and cutaneous horns [4]. *Kaaram*'s caustic properties result in the elimination of unwanted tissues. It induces sterile inflammation at the site of the wart and functions locally as a sclerosing agent. The hyperpigmented papules subsequently detach due to the sclerosis process. *Kaaram *possesses the ability to do precise and localised cutting and healing. *Pachaieruvai*, apart from being a preferred remedy for producing *kaaram,* has also been employed in the treatment of warts, haemorrhoids, and various other ailments.

## Case presentation

Background

A 70-year-old woman with Fitzpatrick skin type V appeared with many hyperpigmented papules on her malar area and adjacent areas that had been present for five years. The patient's previous medical history and family history did not reveal any notable findings. She has not undergone any prior therapy.

Clinical examination

The dermatological examination revealed many brown papular lesions, which were particularly pronounced in the malar areas. The lesions typically varied in size from 1 to 5 mm; in the malar area, they could reach up to 1 cm in size.

Siddha treatment

This therapy was administered with the necessary prerequisite of written consent. *Pachaieruvai* was administered externally for three consecutive days. The evaluation of treatment effectiveness was determined using a standardised quartile grading system: grade 1 (25%; limited to no improvement), grade 2 (26-50%; moderate improvement), grade 3 (51-75%; major improvement), and grade 4 (>75%; practically complete improvement). After treatment, that has shown complete improvement (grade 4).

**Figure 1:**
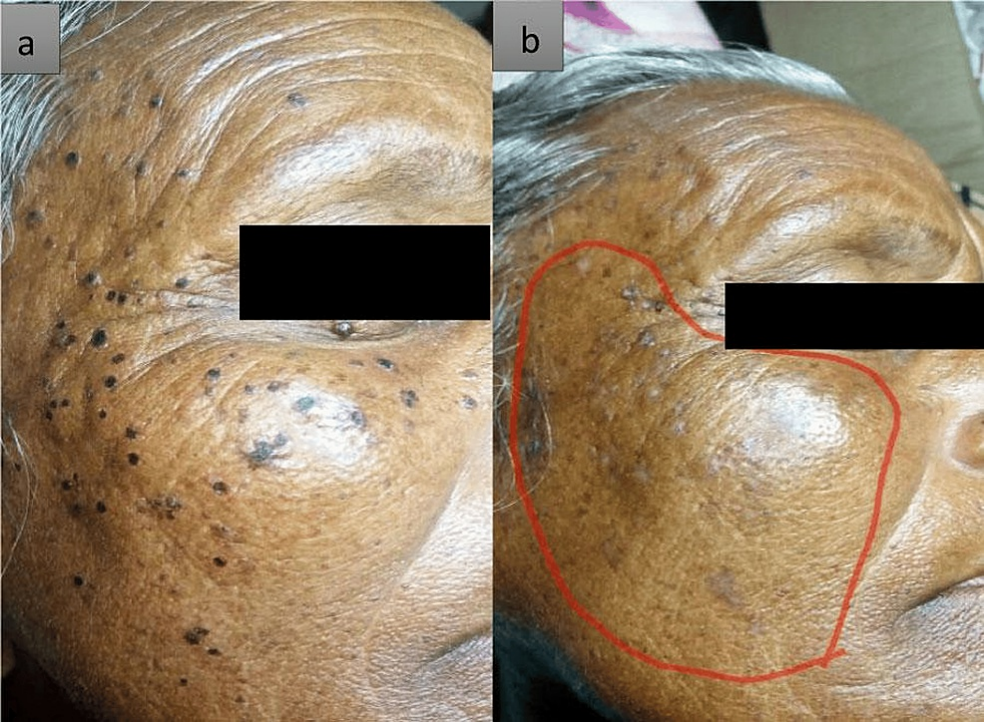
a. Skin lesions on the face before treatment. b. Skin lesions on the face 15 days after treatment.

While undergoing therapy, the patient experienced mild pain, burning, slight redness, and swelling at the area where *Pachaieruvai* was applied. These symptoms persisted for about an hour during and after the procedure. The progress of skin lesions of the face before (a) and 15 days after treatment (b) (Figure 1). At the three-month follow-up visit following the last treatment, grade 4 improvement in all the lesions was noted, with no post-procedural complications or recurrence.

## Discussion

This study provides an initial examination of the management of DPN utilising the *Pachaieruvai*, a traditional Siddha medicine, as described in the existing literature. The composition of *Pachaieruvai* included *vellaipadanam* (arsenic trioxide), *aridharam* (arsenic trisulphide), *thurusu* (copper sulphate), *karchunnam* (calcium carbonate), and *kungiliyam* (resin of Shorea robusta). The utilisation of *Pachaieruvai* in this approach offers some benefits like cost effective, time efficient, minimal dosage, and precise localised effect in comparison to other modalities of the *kaaram* application. *Kaaram* is generally used to excise the unwanted growth or dermophytes. It necessitates a minimal dosage and exhibits precise, localised effects. Furthermore, it is time-efficient, and the number of sessions is restricted. Furthermore, its affordability makes it a profitable choice.

Research has discovered that *vellaipaadanam*, also known as arsenic trioxide, which is a significant component of *Pachaieruvai*, exhibits anti-glioma activity. It has been reported to limit the growth of glioma cells and promote programmed cell death. The activity of promoting the shedding of dead skin cells [5,6,7]. *Aritharam*, also known as arsenic trisulphide, has cytotoxic properties by inhibiting the development and growth of solid tumours [8]. Activity that inhibits or suppresses cell proliferation in *thurusu*, also known as copper sulphate, has a cytotoxic impact that hinders the growth of tumours [9]. It exerts cytotoxic effects through the induction of reactive oxygen species (ROS) production [10]. *Karchunnam*, also known as calcium carbonate, exhibits cytotoxic properties [11]. Similarly, red *kungiliyam*, which is the resin of *Shorea robusta*, has been demonstrated to have cytotoxic effects that inhibit cell proliferation [12].

Mode of action

The suppression of cellular proliferation by the components of *Pachaieruvai* may be attributed to cellular apoptosis. In the discussion section, I provided a clear description of the effects of the components.

Case benefits

The hyperpigmented papules covering the malar region and its surrounding areas were destroyed. During the three-month follow-up visit after the final treatment, it was observed that there was a significant improvement (grade 4) in all the lesions, and there were no adverse effect or recurrence after the procedure.

Limitations of the study

Case studies mainly concentrate on individual patients, which poses challenges in extrapolating findings to larger populations. The presentation and severity of DPN might exhibit significant variation among people, thereby making it inappropriate to generalise conclusions from a single case to all individuals with the illness. Case studies can identify connections between causes and outcomes, but they cannot demonstrate a cause-and-effect relationship. Without controlled trials or extensive observational research, it is challenging to ascertain if specific circumstances directly impact the development or advancement of DPN. Due to the limited scope of case studies, they may not encompass the complete range of DPN manifestations or treatment responses.

Further research is required to comprehensively understand the extent of the condition's variability and evaluate the efficacy of various therapies. There is a higher probability of publishing case studies that exhibit uncommon or dramatic findings, which may introduce a bias towards extreme or atypical situations within the literature. This phenomenon could provide a deceptive perception regarding DPN's frequency and usual progression. Case studies can still help learn about therapy, develop ideas for future research, and show how difficult it can be to manage DPN in real life, even with these limitations. Nevertheless, it is essential to use caution when interpreting these findings and to supplement them with information derived from alternative study designs to understand the phenomenon entirely.

## Conclusions

The response of treatment was assessed based on an established quartile grading scale. After treatment, grade 4 improvement was noted in all the lesions. The hyperpigmented papules covering the malar region and its surrounding areas were destroyed. It can be concluded that the external application with *Pachaieruvai,* a traditional siddha medicine, is effective in treating DPN. *Pachaieruvai* is cost effective and well-tolerated. This case study highlighted the importance of Siddha medicine in treating DPN. No adverse effects that were associated with the medication that was prescribed were noted. However, more studies are needed to confirm our data and to better clarify the possible key role of *Pachaieruvai* in the management of DPN. It is necessary to do additional research on a larger sample size in order to establish the fact that Siddha medications provide an effective method for the management of DPN.
